# Acute Blast Injury Reduces Brain Abeta in Two Rodent Species

**DOI:** 10.3389/fneur.2012.00177

**Published:** 2012-12-21

**Authors:** Rita De Gasperi, Miguel A. Gama Sosa, Soong Ho Kim, John W. Steele, Michael C. Shaughness, Eric Maudlin-Jeronimo, Aaron A. Hall, Steven T. DeKosky, Richard M. McCarron, Madhusoodana P. Nambiar, Sam Gandy, Stephen T. Ahlers, Gregory A. Elder

**Affiliations:** ^1^Research and Development Service, James J. Peters Department of Veterans Affairs Medical CenterBronx, NY, USA; ^2^Department of Psychiatry, Mount Sinai School of MedicineNew York, NY, USA; ^3^Friedman Brain Institute, Mount Sinai School of MedicineNew York, NY, USA; ^4^Department of Neurology, Mount Sinai School of MedicineNew York, NY, USA; ^5^Laboratory of Molecular and Cellular Neuroscience, The Rockefeller UniversityNew York, NY, USA; ^6^Department of Neurotrauma, Operational and Undersea Medicine Directorate, Naval Medical Research CenterSilver Spring, MD, USA; ^7^Department of Neurology, University of Virginia School of MedicineCharlottesville, VA, USA; ^8^Blast-Induced Neurotrauma Branch, Center for Military Psychiatry and Neuroscience, Walter Reed Army Institute of ResearchSilver Spring, MD, USA; ^9^Neurology Service, James J. Peters Department of Veterans Affairs Medical CenterBronx, NY, USA

**Keywords:** abeta, amyloid precursor protein, β-site APP cleaving enzyme 1, blast, mouse, presenilin-1, rat, traumatic brain injury

## Abstract

Blast-induced traumatic brain injury (TBI) has been a major cause of morbidity and mortality in the conflicts in Iraq and Afghanistan. How the primary blast wave affects the brain is not well understood. In particular, it is unclear whether blast injures the brain through mechanisms similar to those found in non-blast closed impact injuries (nbTBI). The β-amyloid (Aβ) peptide associated with the development of Alzheimer’s disease is elevated acutely following TBI in humans as well as in experimental animal models of nbTBI. We examined levels of brain Aβ following experimental blast injury using enzyme-linked immunosorbent assays for Aβ 40 and 42. In both rat and mouse models of blast injury, rather than being increased, endogenous rodent brain Aβ levels were decreased acutely following injury. Levels of the amyloid precursor protein (APP) were increased following blast exposure although there was no evidence of axonal pathology based on APP immunohistochemical staining. Unlike the findings in nbTBI animal models, levels of the β-secretase, β-site APP cleaving enzyme 1, and the γ-secretase component presenilin-1 were unchanged following blast exposure. These studies have implications for understanding the nature of blast injury to the brain. They also suggest that strategies aimed at lowering Aβ production may not be effective for treating acute blast injury to the brain.

## Introduction

Blast-induced brain injury has been of longstanding interest in military head trauma (Jones et al., 2007). Recently, there has been renewed interest in blast related traumatic brain injury (TBI) because of the frequency of blast injury in the conflicts in Iraq and Afghanistan (Elder et al., 2010). How the primary blast wave itself affects the brain (as differentiated from the deceleration injury where the brain is injured by a person hitting an object, such as a wall or the ground) is not well understood (Cernak and Noble-Haeusslein, 2010). Direct tissue damage, bleeding, and diffuse axonal injury (DAI) are the best known pathophysiological mechanisms associated with the type of blunt impact injuries that occur in most non-blast closed impact injuries (nbTBI; Gennarelli and Grahm, 2005). However, whether blast injures the brain through mechanisms similar to those found in nbTBI is unknown.

Several proteins associated with neurodegenerative diseases accumulate in brain following nbTBI, including α-synuclein, tau, the amyloid precursor protein (APP), and its product the β-amyloid (Aβ) protein (Uryu et al., 2007). Accumulation of Aβ protein is most associated with the development of Alzheimer’s disease (AD), and there has been much interest in whether its upregulation following TBI may explain the epidemiological association between a history of prior TBI and the subsequent development of AD (DeKosky et al., 2010; Johnson et al., 2010). Indeed, changes in Aβ occur rapidly after acute TBI; diffuse cortical Aβ deposits and increased levels of soluble Aβ have been observed in humans as early as 2 h after a severe TBI (Ikonomovic et al., 2004; DeKosky et al., 2007, 2010; Johnson et al., 2010). There is also a well-documented synergy between TBI and outcome of TBI in carriers of the apolipoprotein E ε4 allele (Mayeux et al., 1995; Zhou et al., 2008), a connection that suggests some role for Aβ in TBI outcome.

Elevations of Aβ have also been seen acutely in many (Smith et al., 1998; Abrahamson et al., 2009; Loane et al., 2009, 2011; Tran et al., 2011; Tian et al., 2012; Yu et al., 2012; Zhang et al., 2012) although not all (Schwetye et al., 2010) experimental animal models of nbTBI, along with increased expression of components of the γ-secretase complex as well as β-site APP cleaving enzyme 1 (BACE1), the principal β-secretase (Blasko et al., 2004; Chen et al., 2004; Nadler et al., 2008; Loane et al., 2009; Zohar et al., 2011). These observations are all consistent with increased processing of APP (producing increased Aβ) after TBI. Studies in nbTBI animal models also consistently reveal increased APP expression acutely following TBI (Murakami et al., 1998; Van Den Heuvel et al., 2000; Ciallella et al., 2002; Chen et al., 2004).

All prior studies examining Aβ levels in experimental animals have been performed using models that mimic the types of contusional and diffuse brain injuries associated with nbTBI closed impact head injury (Abrahamson et al., 2009; Loane et al., 2009, 2011; Schwetye et al., 2010; Tran et al., 2011; Tian et al., 2012; Yu et al., 2012; Zhang et al., 2012). Whether blast TBI activates primary and secondary cascades similar to those activated in nbTBI is unknown. In ongoing studies we have been examining the acute effects of blast in rat and mouse models (Wang et al., 2011; Ahlers et al., 2012; Elder et al., 2012). During the course of these studies frozen brain tissue was collected across a range of blast exposures corresponding to a spectrum of mild to severe TBI in the rat and severe TBI in the mouse. Here we took advantage of the availability of this tissue to examine levels of brain Aβ following experimental blast injury.

## Materials and Methods

### Animals

Adult male Long Evans Hooded rats (250–350 g; 10–12 weeks age; Charles River Laboratories International, Inc., Wilmington, MA, USA) or C57BL/6 mice (8–10 weeks old; 22–26 g; Jackson Laboratories, Bar Harbor, ME, USA) were used as subjects. All studies were approved by the Institutional Animal Care and Use Committees of the Naval Medical Research Center, the Walter Reed Army Institute of Research (WRAIR), and the James J. Peters VA Medical Center.

### Blast overpressure exposure

Rats were subjected to overpressure exposure using the WRAIR shock tube which simulates the effects of air blast exposure under experimental conditions. The shock tube has a 12′′ circular diameter and is a 19.5-ft long steel tube divided into a 2.5-ft compression chamber that is separated from a 17-ft expansion chamber. The compression and expansion chambers are separated by polyethylene Mylar™ sheets (Du Pont, Co., Wilmington, DE, USA) that control the peak pressure generated (Chavko et al., 2007; Elder et al., 2010). The peak pressure at the end of the expansion chamber was determined by piezoresistive gages specifically designed for pressure-time (impulse) measurements (Model 102M152, PCB, Piezotronics, Inc., Depew, NY, USA).

Individual rats were anesthetized using an isoflurane gas anesthesia system consisting of a vaporizer, gas lines and valves, and an activated charcoal scavenging system adapted for use with rodents. Rats were placed into a polycarbonate induction chamber, which was closed and immediately flushed with 5% isoflurane mixture in air for 2 min. Rats were placed into a cone shaped plastic restraint device and then placed in the shock tube. Movement was further restricted during the blast exposure using 1.5 cm diameter flattened rubber tourniquet tubing. Three tourniquets were spaced evenly to secure the head region, the upper torso, and lower torso while the animal was in the plastic restraint cone. The end of each tubing was threaded through a toggle and run outside of the exposure cage where it was tied to firmly affix the animal and prevent movement during the blast overpressure exposure without restricting breathing. Rats were assigned randomly to sham or blast condition without any body shielding, resulting in full body exposure to the blast wave with the head, upper torso, and lower torso fixed in a plastic restraint cone. Two body orientations were tested – with the rat’s head facing toward or sideways (at a right angle to the wave, counterbalanced left and right) to the blast wave. Blast exposed animals received single 36.6 (*n* = 15), 74.5 (*n* = 26), or 116.7 (*n* = 19) kilopascal (kPa) exposures. Sham exposed animals (*n* = 9) were treated identically except that they did not receive a blast exposure. Mice (*n* = 6 blast and 6 control) were treated in a similar manner and received a single 147 kPa blast exposure with the head perpendicular to the direction of the blast wave in a prone position (Wang et al., 2011).

### Enzyme-linked immunosorbent assays for abeta

Animals were euthanized by CO_2_ narcosis or decapitation after isoflurane anesthesia and the brains were quickly removed, frozen, and stored at −80°C until use. Triton X-100 fractions from one hemisphere in the case of rats, or whole brain in the case of mice, were prepared using a protocol adapted from that described in Kawarabayashi et al. (2001) and described in more detail by Steele et al. (2009). The tissues were homogenized with a hand held homogenizer in 50 mM Tris-HCl buffer, pH 7.4, 150 mM NaCl, 1% Triton X-100 buffer supplemented with a protease/phosphatase inhibitor cocktail (Sigma Aldrich, St. Louis, MO, USA) at a concentration of 0.2 mg fresh tissue/ml of buffer. The homogenates were centrifuged at 100,000 × *g* for 1 h at 4°C. The supernatants were decanted and Aβ 40 and 42 levels were determined by Enzyme-linked immunosorbent assays (ELISAs) using a commercially available kit that detects rodent Aβ (Wako, Richmond, VA, USA). The pellets were then extracted in the above buffer containing 0.25% Na deoxycholate and 0.5% SDS, centrifuged and the supernatant saved (SDS extract).

### Western blotting

Protein samples (Triton X-100 extracts for APP and BACE1; SDS extracts for presenilin-1) were separated by SDS-PAGE and blotted onto polyvinylidene difluoride (PVDF) membranes (Millipore Corporation, Billerica, MA, USA). The primary antibodies utilized were a mouse monoclonal anti-APP (1:1500; Mab348, Millipore Corp.), a rabbit polyclonal anti-BACE1 (1:1500; Millipore Corp.), a rabbit polyclonal anti-β-tubulin antibody (1:4000; Abcam, Cambridge, UK) and the mouse monoclonal antibody 33B10 against the C-terminal fragment of presenilin-1 (1:2000; gift of Dr. Nikolaos Robakis, Mount Sinai School of Medicine, New York, NY, USA). The membranes were probed with the appropriate HRP-conjugated secondary antibodies (GE Life Sciences, Piscataway, NJ, USA), and the protein bands were visualized with the ECL Prime reagent (GE Life Sciences). For reprobing, the membranes were treated with Re-Blot Plus strong stripping solution (Millipore Corp.) according to the manufacturer’s instructions. Quantification was performed using Image Quant TL software (GE Life Sciences). Levels of the target proteins were normalized to levels of β-tubulin.

### Immunohistochemistry

Rats were perfused with 4% paraformaldehyde in phosphate buffered saline (PBS). The brains were dissected and cut into 50 μm thick sections using a Vibratome (Leica, Wetzlar, Germany). Immunohistochemical staining was performed as previously described (Gama Sosa et al., 2010) using a rabbit anti-APP antibody APP369 (1:700), which recognizes the APP C-terminus and detects full-length APP and C-terminal fragments of APP (Keilani et al., 2012) and SMI-31, a mouse monoclonal antibody that recognizes phosphorylated epitopes on the mouse mid-sized and heavy neurofilament proteins (1:500; Covance, Denver, PA, USA).

### Statistical analysis

Depending on the experiment, statistical tests employed univariate or factorial analysis of variance (ANOVA), or unpaired *t*-tests. Equality of variance was first assessed using the Levene test. When the Levene test was not significant (comparisons yielding *p* > 0.05), between-group comparisons were made using unpaired *t*-tests (Student’s), and univariate or factorial ANOVA. *Post hoc* comparisons following univariate ANOVA were performed using Dunnett’s test with sham exposed controls treated as the reference group. When the Levene statistic was significant (*p* < 0.05), unpaired *t*-tests were employed using the Welch correction for unequal variances. Statistical tests were performed using SPSS 20.0 (SPSS, Chicago, IL, USA) or GraphPad Prism 5.0 (GraphPad Software, San Diego, CA, USA).

## Results

### Decreased levels of Aβ in rats exposed to blast overpressure injury

**Figure 1:**
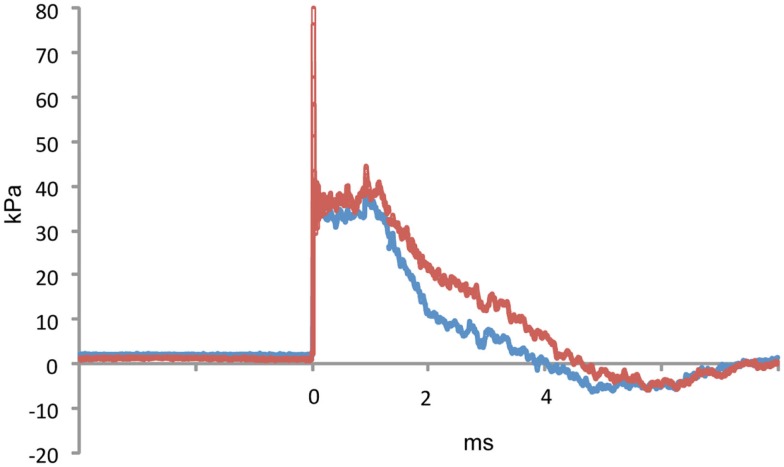
**A representative tracing of a 36.6 kPa blast wave exposure is shown**. The red line indicates dynamic pressure and the blue line static pressure. The blast overpressure duration (ms) was 4.1 ± 0.3 (SEM) and the overpressure integral (kPa*ms) was 75.2 ±4.5. Characteristics of the 74.5 and 116.7 kPa blast waves can be found in Ahlers et al. (2012).

**Figure A1:**
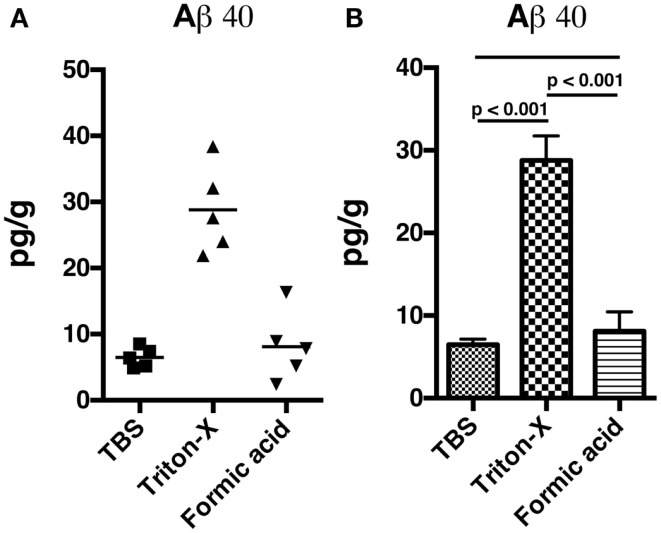
**Non-blast exposed control rats were sacrificed and hemibrains were fractioned into Tris buffered saline soluble, Triton X-100 soluble, and formic acid-extractable fractions**. Levels of Aβ 40 **(A,B)** were determined by ELISA. Values are presented the ±SEM. Univariate ANOVA indicated that Aβ 40 levels differed between the fractions (*F*_2, 12_ = 4.936; *p* < 0.001). Post-test comparisons between the groups are indicated (Tukey’s multiple comparisons test). NS indicates not significant. Note that most Aβ 40 is found in the Triton X-100 soluble fraction.

We utilized a rat model of TBI in which adult male rats received single 36.6, 74.5, or 116.7 kPa exposures. The physical characteristics of the blast wave have been previously described (Ahlers et al., 2012) and a representative tracing of a 36.6 kPa blast wave exposure is shown in Figure 1. Two body orientations were tested with the rats facing toward or sideways (counterbalanced left and right) to the blast wave with the head and body fixed in a plastic restraint cone to restrict movement. Rats were sacrificed either 24 h or 1 week after receiving the blast exposure and Triton X-100 fractions were prepared from one hemisphere using a protocol adapted from Kawarabayashi et al. (2001). In preliminary studies we found that most rat Aβ 40 and 42 were found in the Triton X-100 soluble fraction, compared to the Tris buffered saline and formic acid-extractable fractions (Figure A1 in Appendix). Therefore, for these studies, the Triton X-100 fraction was analyzed.

**Figure 2:**
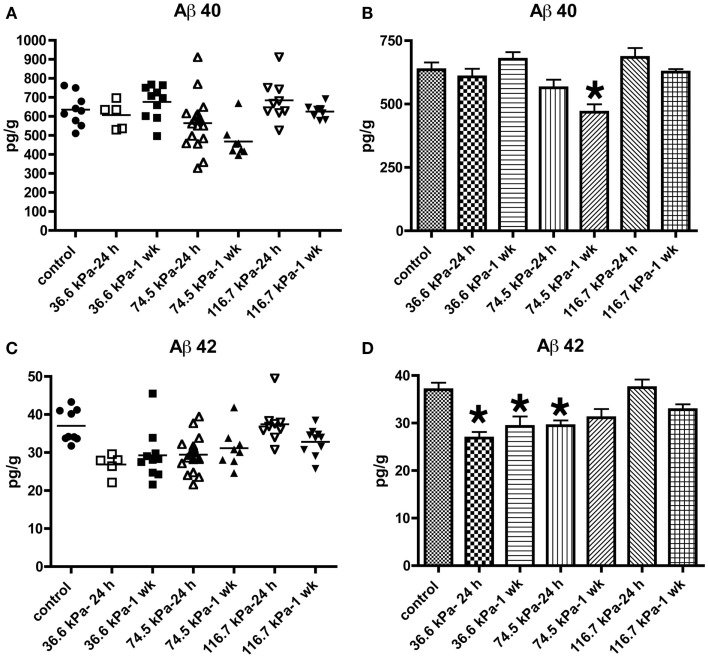
**Enzyme-linked immunosorbent assays for Aβ 40 (A,B) and 42 (C,D) were performed on Triton X-100 brain extracts from rats that were subjected to blast exposures of 36.6, 74.5, or 116.7 kPa and harvested at 24 hours (24 h) or 1 week (1 wk) post-blast exposure**. Controls consisted of sham exposed rats harvested at 24 h (*n* = 4) or 1 week (*n* = 5) post-exposure. Because neither Aβ 40 (*p* = 0.29, unpaired *t*-test) nor 42 (*p* = 0.84) levels differed between the controls harvested at 24 h and 1 week, the two groups were pooled and treated as a single control group (*n* = 9). Numbers of samples in each group are indicated in Table 1. Values are presented ± the SEM. Asterisk indicates values that were significantly different from controls based on Dunnett’s test. Note the decrease in Aβ 42 in rats exposed to a 36.6 kPa blast at 24 h and 1 week post-exposure as well as 74.5 kPa exposed rats at 24 h. By contrast Aβ 40 was decreased in only the 74.5 kPa exposed rats at 1 week. Further statistical comparisons are discussed in the text and presented in Table 1.

Initially we examined all body orientations to blast together in one pooled analysis. A univariate ANOVA showed that Aβ 42 levels were significantly different from controls (*F*_6, 62_ = 5.911; *p* < 0.001). The effect of acute blast exposure on brain Aβ levels at each blast level is shown in Figure 2 and Table 1. Compared to sham exposed controls, the 36.6 kPa exposure at 24 h (*p* = 0.002, Dunnett’s test) and 1 week (*p* = 0.005) as well as the 74.5 kPa exposure at 24 h (*p* = 0.002) resulted in significantly lower levels of Aβ 42. In the 74.5 kPa exposure at 1 week, the level of Aβ 42 was decreased when compared to the control using an unpaired *t*-test (*p* = 0.02) although this decrease did not reach statistical significance when multiple comparisons were corrected for using Dunnett’s test (*p* = 0.067). By contrast, the highest-level exposure (116.7 kPa) did not affect levels of brain Aβ 42 at 24 h and affected Aβ 42 at 1 week only if multiple statistical comparisons were ignored (Table 1). While a univariate ANOVA indicated that Aβ 40 was also affected following blast exposure (*F*_6, 61_ = 4.936; *p* < 0.001), pairwise comparison of each experimental blast condition to a matched control revealed that only the 74.5 exposure at 1 week was significantly lower than the control (*p* = 0.006 Dunnett’s test).

**Table 1:** **Levels of abeta 40 and 42 in blast exposed rats**.

	*N*	Mean	SEM	SD	Dunnett’s test vs. control	Unpaired *t*-test vs. control
**Aβ 40**						
Control	9	635.5	28.27	84.82	NA	NA
36.6 kPa-24 h	5	607.0	31.88	71.28	0.99	0.53
36.6 kPa-1 week	10	676.6	28.00	88.54	0.88	0.31
74.5 kPa-24 h	18	564.2	31.83	135.0	0.32	0.16
74.5 kPa-1 week	8	468.1	31.04	87.79	0.006	0.001
116.7 kPa-24 h	9	684.3	36.37	109.1	0.80	0.30
116.7 kPa-1 week	9	625.8	11.78	35.35	1.00	0.75
**Aβ 42**						
Control	9	37.03	1.437	4.311	NA	NA
36.6 kPa-24 h	5	26.84	1.273	2.846	0.002	0.0005
36.6 kPa-1 week	10	29.29	2.096	6.627	0.005	0.008
74.5 kPa-24 h	18	29.46	1.086	4.606	0.002	0.0004
74.5 kPa-1 week	8	31.13	1.828	5.174	0.067	0.02
116.7 kPa-24 h	9	37.44	1.698	5.095	1.00	0.85
116.7 kPa-1 week	10	32.81	1.146	3.623	0.24	0.03

**N*, number; NA, not applicable; SEM, standard error of the mean; SD, standard deviation*.

### No effect of orientation to the blast wave on Aβ levels in rats exposed to blast overpressure injury

**Figure 3:**
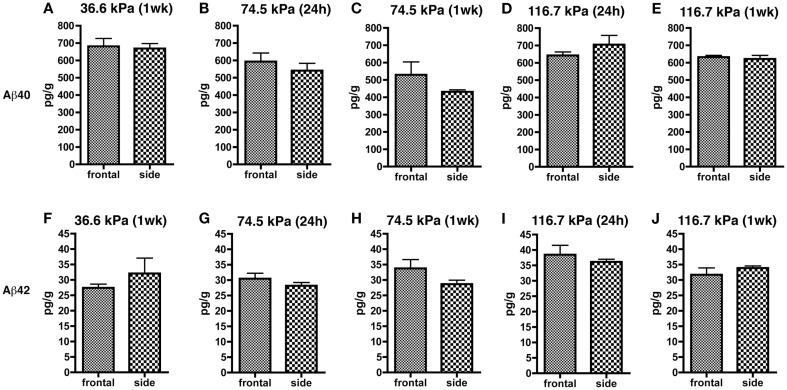
**Results of Aβ 40 (A–E) and 42 (F–J) ELISAs on Triton X-100 brain extracts are presented for rats exposed to frontal or side blast exposures of 36.6 (A,F), 74.5 (B,C,G,H), or 116.7 kPa (D,E,I,J) harvested at 24 hours (24 h) or 1 week (1 wk) post-blast exposure**. Values are presented ± the SEM. There were no statistically significant differences between any of the pair wise comparisons (unpaired *t*-tests) demonstrating that orientation to the blast had no effect on levels of Aβ 40 or 42. Further statistical tests are discussed in the text. Sample sizes were: **(A,F)** (6 frontal and 4 side), **(B,G)** (8 frontal and 10 side), **(C,H)** (3 frontal and 5 side), **(D,I)** (5 frontal and 4 side), and **(E,J)** (4 frontal and 5 side).

Since orientation to the blast wave might affect the brain’s response due to alignment of long tracts or structural discontinuities, we compared both frontal and side/lateral/perpendicular exposures (Figure 3). A two-way ANOVA using blast exposure and orientation as fixed variables did not reveal any interaction effect of wave direction and orientation for either Aβ 40 (*F*_4, 44_ = 0.979; *p* = 0.42) or 42 (*F*_4, 45_ = 1.102; *p* = 0.36). Pairwise comparisons (unpaired *t*-tests) revealed no differences between the frontal and side (lateral) orientations at any of the blast pressures tested, for either Aβ 40 or 42. Thus, head/skull orientation to the blast wave does not appear to affect the Aβ response in brain.

### Increased levels of APP but no evidence for abnormal APP staining of axons in rats exposed to blast overpressure injury

**Figure 4:**
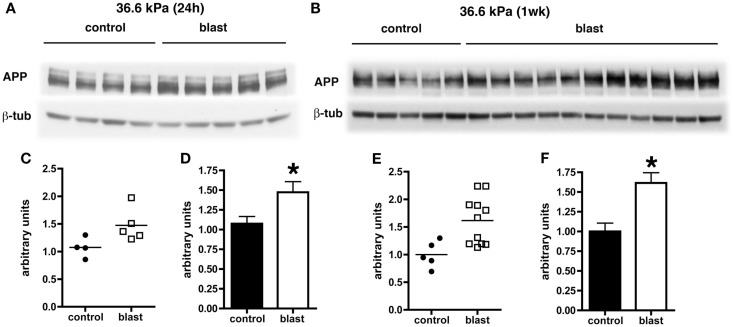
**Western blotting was performed on hemi brain Triton X-100 extracts from sham exposed (controls) or rats exposed to 36.6 kPa blast injury harvested at 24 h (A) or 1 week post-blast exposure (B)**. The top panel in each set shows blotting with an antibody that recognizes APP. In the lower panels the blots were reprobed for β-tubulin (β-tub) as a loading control. Levels of APP are expressed as the ratio of APP to β-tubulin (±SEM) for the experiments in **(A)** in **(C,D)** and for **(B)** in **(E,F)**. Asterisk indicates *p* = 0.05 or less vs. control (unpaired *t*-test). Note the increase in APP levels at both 24 h and 1 week post-blast exposure.

Levels of APP expression increase acutely after experimental TBI in rodents (Loane et al., 2009; Tian et al., 2012; Zhang et al., 2012). To determine whether blast affects expression of APP, we examined APP levels by quantitative Western blotting. Since the most consistent changes in Aβ 42 were in the 36.6 kPa exposures, we compared 36.6 kPa blast exposure to their paired (non-blast exposed) controls. We found APP levels were higher in blast exposed animals both 24 h (*p* = 0.05, unpaired *t*-test) and 1 week post-exposure (*p* = 0.01; Figure 4). Thus, blast exposure increased APP levels despite reductions in Aβ 42.

**Figure 5:**
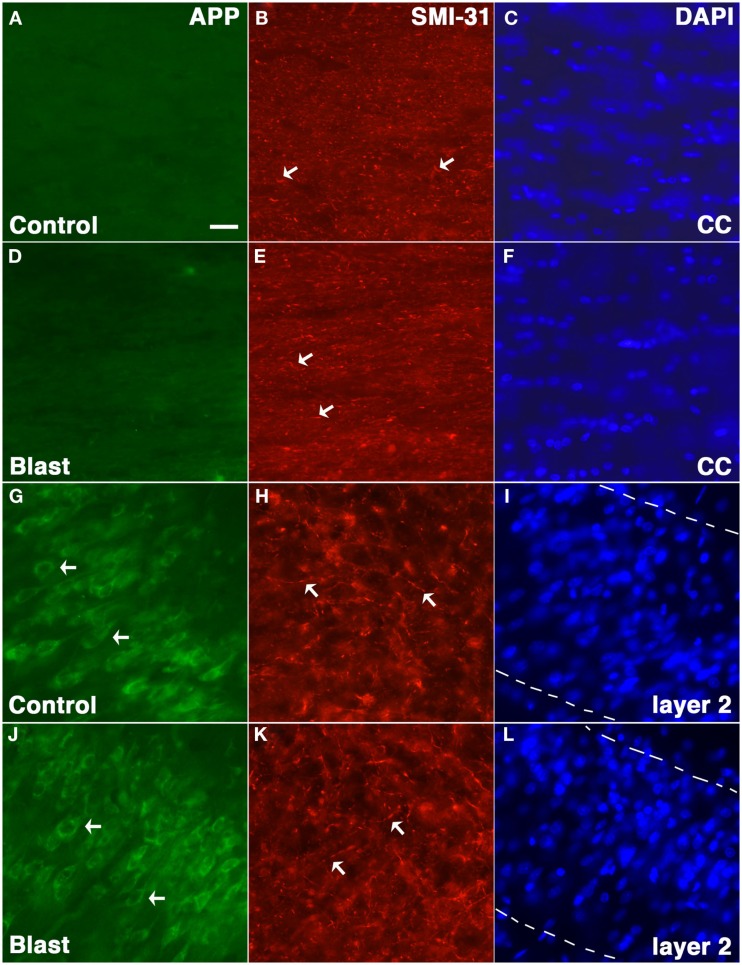
**Rats were exposed to 74.5 kPa or control (i.e., sham) conditions and were sacrificed 24 h later**. Sections from control **(A–C,G–I)** and blast exposed **(D–F,J–L)** were immunostained with the rabbit anti-APP antibody 369 **(A,D,G,J)** directed against the APP C-terminus (green) and SMI-31 **(B,E,H,K)** a mouse monoclonal antibody which recognizes phosphorylated epitopes on the mouse mid-sized and heavy neurofilament proteins that are found mostly in axons (red). Sections counterstained with a DAPI nuclear stain (blue) are shown in **(C,F,I,L)**. Staining in the corpus callosum **(A–F)** and layer 2 of the piriform cortex **(G–L)** is shown. Note the lack of APP staining in the corpus callosum (CC) in either blast exposed or control and the fine axonal staining with SMI-31 which is indicated by arrows in **(B,E,H,K)** that is present in both blast and control. Staining of neurons (arrows) in layer 2 of the piriform cortex is shown as a positive control for staining with the anti-APP antibody **(G,J)**. The margins of layer 2 are indicated by broken lines in **(I,L)**. Scale bar: 10 μm.

Amyloid precursor protein accumulation in axons is widely used as a marker of axonal injury in both humans and experimental animal models of nbTBI (Gentleman et al., 1993; Sherriff et al., 1994; Lewen et al., 1995; Pierce et al., 1996; Stone et al., 2000). To determine whether abnormal axonal staining was present we performed APP immunostaining on tissue harvested from blast exposed rats at 24 h following a 74.5 kPa exposure comparing these to sham exposed controls. Neither controls nor blast exposed rats showed any evidence of axonal staining with APP (Figure 5).

### Unchanged levels of BACE1 and presenilin-1 in rats exposed to blast overpressure injury

**Figure 6:**
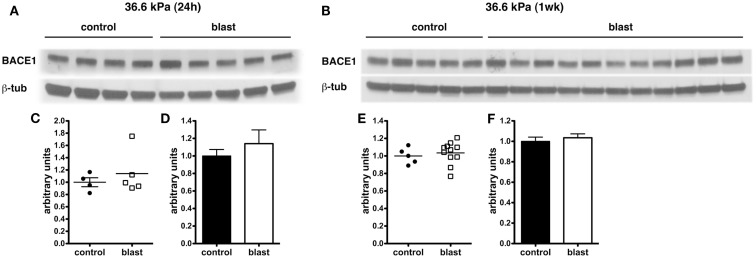
**Western blotting was performed on hemi brain Triton X-100 extracts from sham exposed (controls) or rats exposed to 36.6 kPa blast injury harvested at 24 h (A) or 1 week post-blast exposure (B)**. The top panel in each set shows blotting with an antibody that recognizes BACE1. In the lower panels the blots were reprobed for β-tubulin (β-tub) as a loading control. Levels of BACE1 are expressed as the ratio of BACE1 to β-tubulin (±SEM) for the experiments in **(A)** in **(C,D)** and for **(B)** in **(E,F)**. Note the lack of change in BACE1 levels in rats exposed to a 36.6 kPa blast at either 24 h or 1 week.

**Figure 7:**
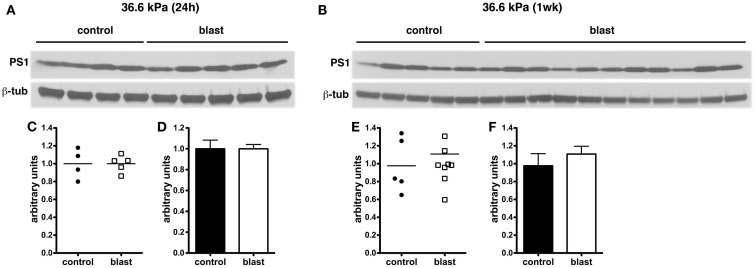
**Western blotting was performed on hemi brain SDS extracts from sham exposed (controls) or rats exposed to 36.6 kPa blast injury harvested at 24 h (A) or 1 week post-blast exposure (B)**. Blotting was performed with an antibody that recognizes presenilin-1 (PS1) and the blots were reprobed for β-tubulin (β-tub). Levels of presenilin-1 are expressed as the ratio of presenilin-1 to β-tubulin (±SEM) for the experiments in **(A)** in **(C,D)** and for **(B)** in **(E,F)**. Note the lack of change in PS1 levels in rats exposed to a 36.6 kPa blast at either 24 h or 1 week.

In experimental animal models of nbTBI, BACE1 (Blasko et al., 2004; Chen et al., 2004; Loane et al., 2009; Zohar et al., 2011) along with presenilin-1 (Chen et al., 2004; Nadler et al., 2008; Loane et al., 2009), a component of the γ-secretase complex, have been reported to be increased (Blasko et al., 2004; Chen et al., 2004; Nadler et al., 2008; Loane et al., 2009; Zohar et al., 2011). However, we did not find any changes in levels of either BACE1 (Figure 6) or presenilin-1 (Figure 7) by Western blotting in rats subjected to 36.6 kPa blast exposures at 24 h or 1 week after exposure.

### Acute decreases in levels of Aβ in mice exposed to blast overpressure injury

**Figure 8:**
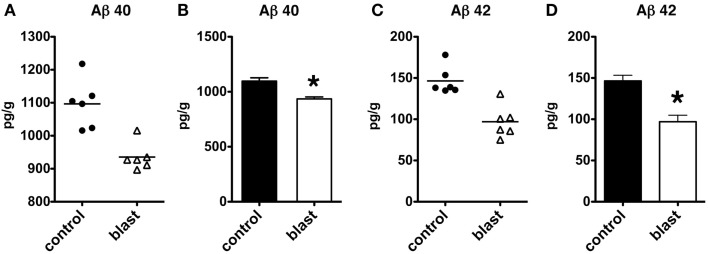
**Enzyme-linked immunosorbent assays for Aβ 40 (A,B) and 42 (C,D) were performed on total brain extracts from mice exposed to blast injuries of 147 kPa and harvested at 24 h post-blast exposure**. Controls consisted of sham exposed mice. Values are presented the ±SEM. Asterisk indicates values that were significantly different from controls (unpaired *t*-tests). Note the reduction in Aβ 40 and 42.

We next determined whether similar changes might be found in another rodent species. C57BL/6 mice were subjected to a single 147 kPa exposure, and the brains were harvested 24 h after exposure. Both Aβ 40 (*p* < 0.001, unpaired *t*-test) and Aβ 42 (*p* < 0.001) were significantly decreased in blast exposed mice, thus showing that lowering of Aβ is likely a general response in rodent species to acute blast (Figure 8).

## Discussion

Epidemiological studies support an association between single incident severe TBI and the later development of AD (DeKosky et al., 2010; Johnson et al., 2010). A variety of studies in humans as well as experimental animals have documented the rapid appearance of Aβ deposits and increased Aβ levels in the setting of acute TBI (Ikonomovic et al., 2004; DeKosky et al., 2007; Abrahamson et al., 2009; Loane et al., 2009, 2011; Tran et al., 2011; Tian et al., 2012; Yu et al., 2012; Zhang et al., 2012) although none of these studies have included subjects with blast injuries.

In this series of studies we determined effects of blast injury on Aβ levels in rodent brain, using both rat and mouse models of blast-induced brain injury. Three blast levels in rats were tested. Importantly prior studies using these models established that exposures up to 74.5 kPa, while representing a blast level that is transmitted to brain (Chavko et al., 2007), lead to no persistent neurological impairments (Ahlers et al., 2012), nor result in gross neuropathological effects or lung pathology (Ahlers et al., 2012). Thus, exposures of 36.6 or 74.5 kPa are consistent with those types of blast that might be associated with mild TBI or subclinical blast exposure. By contrast, moderate to severe TBIs are associated with evident neuropathology as well as significant neurological deficits and indeed 116.7 kPa blast exposures were often associated with transient loss of the righting reflex – which is believed to approximate loss of consciousness in rodents as well as impairment of motor and cognitive function (Ahlers et al., 2012). In addition, approximately 30% of 116.7 kPa blast exposed animals had gross cerebral and subdural hemorrhages as well as contusions (Ahlers et al., 2012) and 116.7 kPa exposures also resulted in significant lung pathology (Chavko et al., 2006, 2007; Ahlers et al., 2012).

In our studies we showed that, in both rats and mice, blast injury leads to diminished levels of Aβ 42 in rats and decreased levels of both Aβ 40 and 42 in mice. Interestingly, the effect on Aβ 42 was most prominent in rats exposed to the lower blast exposures (36.6 and 74.5 kPa), while there were no effects on Aβ 42 at the 116.7 kPa exposure level. In mice, only a single blast exposure of 147 kPa was tested. While it is difficult to directly compare blast exposures in mice and rats, in mice this exposure is typically associated with increased righting reflex time, weight loss, production of reactive oxygen species, motor dysfunction and occasionally mild cerebral hemorrhages and other neuropathology making it more equivalent to a severe TBI (Wang et al., 2011). Such higher blast pressures are also typically associated with lung pathology that may worsen aspects of blast-associated brain injury by effects mediated through the autonomic nervous system (Cernak, 2010). Although, the converse may also be true, in that blast exposure to the brain may contribute to lung pathology (Cernak, 2010).

Although prior studies have shown that a frontal orientation to the blast wave is associated with more acute behavioral effects (Ahlers et al., 2012), there was no effect of a frontal vs. lateral (side) orientation on Aβ levels. There were also no consistent effects on Aβ 40 levels in rats, with only the 74.5 kPa exposure showing diminished levels 1 week post-exposure. While Aβ 40 was also decreased by ≈15% in the one mouse exposure, this decrease was much less than the ≈50% decline seen in Aβ 42, suggesting that in both species the effect of blast is much greater on Aβ 42 levels than on Aβ 40 levels.

Why Aβ typically increases acutely following TBI is not entirely clear. The Aβ peptide itself is derived from processing of APP, the larger precursor protein. In AD, the 39–42 amino acid Aβ peptide deposits in senile plaques (Gandy, 2005). Many *in vitro* and *in vivo* studies have demonstrated that in particular the longer Aβ 42 species can be neurotoxic and that shunting of APP processing toward Aβ 42 production sets off a chain of pathological events (Gandy, 2005). Multiple studies in experimental animals have found that APP expression increases acutely following TBI (Murakami et al., 1998; Van Den Heuvel et al., 2000; Ciallella et al., 2002; Chen et al., 2004). Simultaneously there is increased expression of BACE1 as well as elements of the γ-secretase complex (Blasko et al., 2004; Chen et al., 2004; Nadler et al., 2008; Loane et al., 2009; Zohar et al., 2011) – all of which would predict that there would be increased processing of APP toward Aβ leading to great interest in the possibility that similar neurotoxic mechanisms might be operative in both AD and TBI. However, this would not explain the results here since, despite increased APP levels, in rats subjected to the 36.6 kPa blast exposure levels of Aβ 42 were reduced.

The paradoxical elevation of APP in the setting of reduced Aβ production might be explained by accumulation of unprocessed APP in axons, and indeed, accumulation of APP in axons is used widely as a marker of axonal injury in both humans and experimental animal models of TBI (Gentleman et al., 1993; Sherriff et al., 1994; Lewen et al., 1995; Pierce et al., 1996; Stone et al., 2000). However, while one study has reported APP accumulation in axons following blast exposure (Kuehn et al., 2011), others have not (Garman et al., 2011; Pun et al., 2011; Risling et al., 2011; Ahlers et al., 2012). Risling et al. (2011), for example, noted no APP accumulation in axons of rats exposed to 130 and 260 kPa exposures. Garman et al. (2011) studied rats exposed to greater than 240 kPa (35 psi) blast exposures and found widespread evidence of DAI by silver staining. However, despite evidence of extensive axonal injury by silver staining, APP-stained sections typically showed only minimal axonal staining, except for rats studied at 24 h post-exposure where some mild axonal staining was evident within the deep cerebellar white matter and adjacent to some foci of acute neuronal degeneration. Interestingly, Pun et al. (2011) although not commenting on axonal staining did note more APP positive cells in the white matter of rats at 1 day following blast exposures of 48.9 or 77.3 kPa which are quite similar to those studied here. However, in the present studies we did not observe any axonal staining with APP or any obvious alteration in perikaryal staining in blast exposed rats. Thus pure blast exposure does not appear to induce accumulation of APP in axons in the consistent fashion apparent in nbTBI.

Future studies will be needed to elucidate the mechanism underlying the acute effects of blast exposure on Aβ production. These studies have practical implications for the treatment of acute blast injury, since blocking Aβ production by a variety of pharmacological or genetic means has been reported to reduce tissue damage acutely and improve outcome following controlled cortical impact injuries (CCI) in mice (Abrahamson et al., 2009; Loane et al., 2009, 2011). However, the studies reported here suggest that such strategies may not be applicable to treatment of acute blast injuries.

## Conflict of Interest Statement

The authors have no competing financial interests. The views expressed in this article are those of the authors and do not necessarily reflect the official policy or position of the Department of the Navy, Department of Defense, nor the U.S. Government.
